# Role of Electron Correlation beyond the Active Space in Achieving Quantitative Predictions of Spin-Phonon Relaxation

**DOI:** 10.1021/acs.jctc.4c01696

**Published:** 2025-03-12

**Authors:** Soumi Haldar, Lorenzo A. Mariano, Alessandro Lunghi, Laura Gagliardi

**Affiliations:** † Department of Chemistry, Chicago Center for Theoretical Chemistry, University of Chicago, Chicago, Illinois 60637, United States; ‡ School of Physics, CRANN and AMBER Research Centre, Trinity College, Dublin 2, Ireland

## Abstract

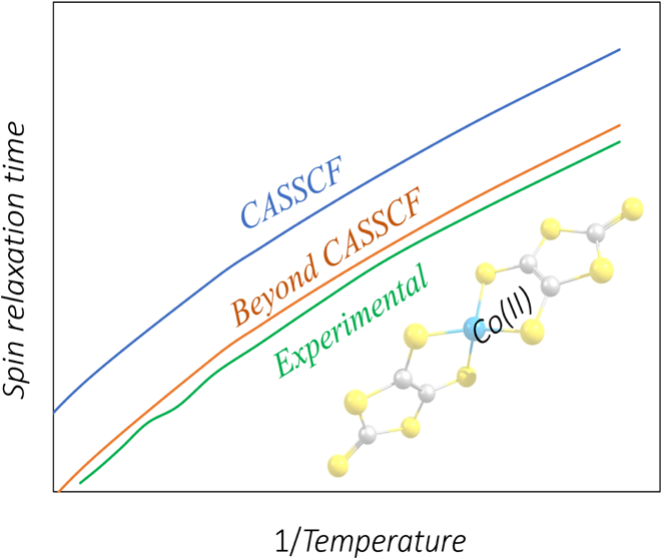

Single-molecule magnets (SMMs) are promising candidates for molecular-scale data storage and processing due to their strong magnetic anisotropy and long spin relaxation times. However, as the temperature rises, interactions between electronic states and lattice vibrations accelerate spin relaxation, significantly limiting their practical applications. Recently, ab initio simulations have made it possible to advance our understanding of phonon-induced magnetic relaxation, but significant deviations from the experiments have often been observed. The description of molecules’ electronic structure has been mostly based on complete active space self-consistent field (CASSCF) calculations, and the impact of electron correlation beyond the active space remains largely unexplored. In this study, we provide the first systematic investigation of spin-phonon relaxation in SMMs with post-CASSCF multiconfigurational methods, specifically CAS, followed by second-order perturbation theory and multiconfiguration pair-density functional theory. Taking Co(II)- and Dy(III)-based SMMs as case studies, we analyze how electron correlation influences spin-phonon relaxation rates across a range of temperatures by comparing theoretical predictions with experimental observations. Our findings demonstrate that post-CASSCF treatments make it possible to achieve quantitative predictions for Co(II)-based SMMs. For Dy(III)-based systems, however, accurate predictions require the consideration of additional effects, underscoring the urgent necessity of further advancing the study of the effects of electronic correlation in these complex systems.

## Introduction

The inherent magnetic bistability exhibited by single-molecule magnets (SMMs) leads to exciting applications of this class of molecules in quantum computing,^1^ magnetic data storage,^2^ and spintronics.^3^ These systems are distinguished by their ability to retain magnetic information at a molecular level at low temperatures, even after removing the magnetizing field, just like bulk hard ferromagnets. The reason they can retain their magnetization for so long is that the doubly degenerate ground magnetic sublevels with opposite spin orientations are separated by a large energy barrier because of which the magnetic reversal or spin flip between the two states is very slow. Having a long enough lifetime, the doubly degenerate spin sublevels can be efficiently used as magnetic binary memory units or quantum bits (qubits). However, in the presence of any interactions with their environment, the performance and functionality of SMMs are drastically reduced by a shortened spin lifetime. At finite temperatures, one of the main sources of such perturbation is the interaction between spins and quantized lattice vibrations known as phonons.^4,5^ Due to such interaction, the electronic or nuclear spins can absorb/emit one or multiple phonons from/into the lattice, eventually bringing spin back to thermal equilibrium. There are multiple possible phonon-involved mechanisms through which magnetic relaxation can take place. At high temperatures, the relaxation proceeds through the Orbach mechanism via a series of sequential absorptions and emissions of high-energy resonant optical phonons.^6,7^ This mechanism shows a characteristic exponential temperature dependence. At low temperatures, however, due to the low population of high-energy phonons, the magnetic relaxation is instead induced by Raman processes involving low-energy phonons.^7,8^ Understanding the interplay between the spin and phonon degrees of freedom and the phonon-assisted relaxation of the magnetic moments in these systems is crucial for the development of high-performance SMMs and their application technologies.

Recent advances have enhanced the understanding of phonon-induced magnetic relaxation in SMMs through first-principles simulations of open quantum systems.^7−14^ Most of these studies utilize multireference electronic structure methods such as the complete active space self-consistent field (CASSCF)^15−17^ to capture the strongly correlated d- or f-element energy landscapes driving SMM magnetic behavior. However, CASSCF neglects electron correlation outside the active space, which can significantly affect predictions both quantitatively and qualitatively.

Some popular post-CASSCF methods are N-electron valence perturbation theory to the second-order (NEVPT2),^18−20^ complete active space second-order perturbation theory (CASPT2),^21,22^ and multiconfiguration pair-density functional theory (MC-PDFT).^23−26^ The latter offers CASPT2-level accuracy at a significantly reduced computational cost.

Ungur and Chibotaru^27^ demonstrated that using CASPT2 improves the theoretical prediction of crystal field splitting in lanthanide complexes compared to CASSCF. For an Er-complex, they found that CASPT2 corrects the CASSCF-computed crystal field spectrum and magnetic properties, aligning computed values more closely with experimental measurements. Neese and co-workers have reported the crucial role of NEVPT2 in refining the CASSCF-computed SMM properties such as spin–orbit splitting, magnetic anisotropy, and spin Hamiltonian parameters in several transition metal-^28−33^ and lanthanide-based^34,35^ SMMs. The impact of going beyond a CASSCF treatment on spin-phonon relaxation dynamics in SMMs, however, remains underexplored. By altering the energy separation of Kramers doublets and influencing spin-phonon coupling strength, a post-CASSCF method could introduce new relaxation channels or modify relaxation time scales. This study is particularly urgent in the face of common deviations up to 1 order of magnitude between experiments and simulations.

**Figure 1:**
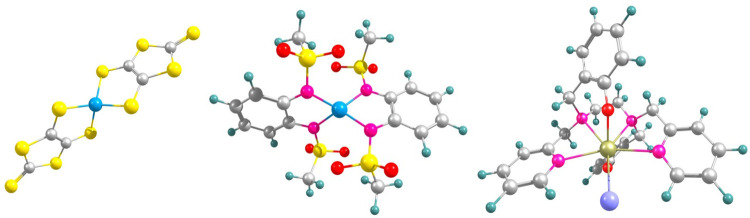
Structure of the SMMs studied in this work. Left: [Co(C_3_S_5_)_2_]^2–^ (**1**), middle: [CoL_2_]^2^–^^ (**2**), and right: [Dy(bbpen)Cl] (**3**). **1** and **2** are shown omitting the counterions; **3** is neutral. Color codes for atoms: Co in blue, Dy in golden, S in yellow, C in gray, N in pink, O in red, Cl in light purple, H in light cyan.

This work marks the first systematic application of CASPT2 and MC-PDFT as electronic structure methods for computing spin relaxation in SMMs. The goal is to provide an accuracy superior to CASSCF, but at a similar cost, if MC-PDFT were the method of choice. As case studies, we explore spin-phonon relaxation dynamics in two mononuclear cobalt(II)-based SMMs, namely [Co(C_3_S_5_)_2_](Ph_4_P)_2_^36^ (**1**) and [CoL_2_][(HNEt_3_)_2_],^37^ where H_2_L = 1,2-bis (methanesulfonamido)benzene (**2**), as well as a Dy(III)-based SMM, namely [Dy(bbpen)Cl],^38^ where H_2_bbpen = *N*,*N*′-(bis(2-hydroxybenzyl) –*N*,*N*′-bis (2-methylpyridyl) ethylenediamine) (**3**). The structures of **1**–**3** (without the counterions) are reported in Figure 1. All three SMMs exhibit long spin relaxation times and have been extensively studied before, representing the ideal testbed for determining the importance of electronic correlation for spin-phonon relaxation predictions.

## Theory

### Electronic Structure Calculations

The SMMs chosen for this study have a multireference nature. Therefore, we employ the state-average complete active space self-consistent field method (which we will refer to as CASSCF) to capture the electron correlation inside the active space (AS). In the following, we will refer to it as static correlation. The CASSCF wave function is constructed as a linear combination of all possible configurations within the active space
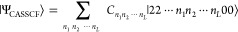
1where the ket vector represents a specific electronic configuration with “2” being the doubly occupied core orbitals, *n*_*i*_ being the occupation number of the *i*th active orbital, and “0” being the unoccupied virtual orbitals. *C*_*n*_1_*n*_2_···*n*_*L*__ is the coefficient for each configuration. The CASSCF energy is expressed as
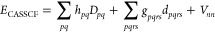
2where *p*, *q*, *r*, and *s* are the general spatial molecular orbital indices. *h*_*pq*_ and *g*_*pqrs*_ are the one- and two-electron integrals, *D*_*pq*_ and *d*_*pqrs*_ are the one- and two-body reduced density matrices, respectively, and *V*_*nn*_ is the sum of the nucleus–nucleus repulsions.

Starting from the reference CASSCF wave function, we perform CASPT2 and MC-PDFT calculations. CASPT2 provides a second-order perturbation correction to the CASSCF energy.^39^ The description of the CASPT2 method can be found in the literature.^21,40−43^

In the MC-PDFT method,^23−26^ the classical energy is obtained from the reference CASSCF wave function, and then, an on-top pair-density functional is used to compute the nonclassical exchange-correlation energy. The total MC-PDFT energy is expressed as
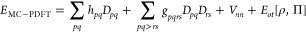
3where the first two terms correspond to the classical energy, and *E*_*ot*_ is the functional of the density (ρ) and the on-top pair-density (Π). Different functional forms can be chosen for *E*_*ot*_, and the computed energies are dependent on the functional forms. The most widely used on-top functional is translated Perdew–Burke–Ernzerhof (tPBE),^23,44^ with densities and density gradients obtained using the PBE functional form. A hybrid functional called tPBE0 mixes 25% of local exchange with the Hartree–Fock exchange. The accuracy of tPBE and tPBE0 has been shown to be similar to CASPT2 for bond energies,^45,46^ spin splitting,^47−49^ and excitation energies.^50,51^ However, the cost of running an MC-PDFT calculation is comparable to the cost of CASSCF.

While CASPT2 computes a second-order correction to the CASSCF energy, usually termed dynamic correlation, MC-PDFT uses the CASSCF wave function to compute the total energy with a functional expression. It is thus not formally correct to say that MC-PDFT recovers a dynamic correlation. In the following, we will thus simply discuss going beyond the CASSCF approximation without distinguishing between static and dynamic correlation because such a distinction makes sense in the CASPT2 case but not in the MC-PDFT case.

### Effective Spin Hamiltonian

In the field of molecular magnetism, it is common practice to describe the magnetic properties of a system using an effective spin Hamiltonian *Ĥ*_*s*_ tailored to represent the ground-state magnetic multiplet.^52,53^ This subspace of the Hilbert space contains the lowest 2*J* + 1 states of the system, and its description through an effective spin Hamiltonian offers the primary advantage of simplifying the interpretation of experimental measurements. Furthermore, when the spin Hamiltonian is derived from *ab initio* electronic structure calculations, it becomes possible to exploit this simplified form of the total electronic Hamiltonian *Ĥ*_*el*_ to efficiently compute couplings between the lattice and the spin degrees of freedom.^13^

In the absence of an external magnetic field, the specific form of a generalized model spin Hamiltonian for a single spin system is expressed as^52^
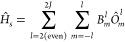
4where the operators *Ô*_*m*_^*l*^ are the tesseral functions of the total angular momentum operators *Ĵ* of rank *l* and order *m. J* is the total angular momentum quantum number, and *B*_*m*_^*l*^ are the spin Hamiltonian parameters that capture the dependence of the magnetic properties on the electronic structure. We extract these spin Hamiltonian parameters at the equilibrium geometry via a mapping between the matrix elements of *Ĥ*_*el*_ and *Ĥ*_*s*_, where *Ĥ*_*el*_ is inclusive of spin–orbit coupling (SOC)
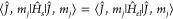
5

It is to be noted that for this mapping in eq 5, the spin Hamiltonian and the electronic Hamiltonian must be expressed in a common basis, the spin eigenstates basis |*Ĵ*,*m*_*j*_⟩. Since the spin Hamiltonian is only defined for the lowest 2*J* + 1 states of the full Hilbert space, the spin basis is obtained by diagonalizing the (2*J* + 1) × (2*J* + 1) block of *Ĵ*_*z*_ expressed in *ab initio* basis and opportunely rotated along the easy axes of magnetization of the system.^54^ The *ab initio* basis is obtained by diagonalizing the electronic Hamiltonian *Ĥ*_*el*_ in the spin-free basis. The *ab initio* basis constructed from these methods is thus different, by virtue of which the spin eigenstates of *Ĵ*_*z*_ are also different for different methods. As a result, the new spin eigenstate basis sets are different for CASSCF, CASPT2, and MC-PDFT. The resulting spin Hamiltonian parameters extracted from eq 5 are therefore also different for different methods, which in turn should be reflected in the computed relaxation time. In this and in the following sections, we use *J* to denote the total angular momentum of the system. However, it is important to note that for both the Co(II)-based compounds we have studied in this work, the orbital angular momentum *L* is at least partially quenched, and a description of the ion’s levels is better performed assuming a spin Hamiltonian, i.e., considering *J* = *S*.^55^ In this case, the Hamiltonian in eq 4 only contains the term with *l* = 2, which is equivalent to the standard zero-field splitting Hamiltonian,
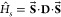
6

### Relaxation Dynamics

In order to simulate the phonon-induced spin relaxation dynamics, the total Hamiltonian of the entire system is constructed by adding the three contributing terms coming from the spin subsystem, the phonon system, and the contribution due to the interaction between these subsystems
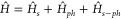
7where *Ĥ*_*s*_ is the model spin Hamiltonian as introduced in eq 4. *Ĥ*_*ph*_ is the component that describes the phonon modes of the system, approximated as a sum of harmonic oscillators
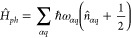
8where *n̂*_*αq*_ is the phonon number operator for a particular phonon mode with frequency ω_*αq*_. The summations run over the phonon mode indices α as well as the reciprocal lattice vectors *q*. Since we consider phonons only at the Γ point, i.e., the center of the Brillouin zone (*q* = (0, 0, 0)), we drop the index *q* for the rest of the discussion.

The last component *Ĥ*_*s*–*ph*_ is the spin-phonon coupling term, which captures the intensity or strength of the interaction of the electronic subsystem with the weakly coupled phonon bath. During the crystal vibrations, the effect of slight changes in nuclear coordinates on the magnetic properties is quite small and can be modeled as perturbations. Therefore, to obtain the coupling term under the weak coupling approximation, the spin Hamiltonian can be expressed as a Taylor expansion around the equilibrium geometry with respect to the nuclear displacements.^5^
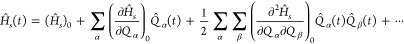
9where the zeroth order term (*Ĥ*_*s*_)_0_ corresponds to the spin Hamiltonian at equilibrium geometry, which appears as the first term in eq 7, and the higher-order terms that explicitly depend on time describe the coupling Hamiltonian *Ĥ*_*s*–*ph*_. As discussed in the previous section, the spin Hamiltonian parameters are highly sensitive to even slight nuclear distortions. In fact, it is at the heart of spin-phonon coupling simulation; the coupling coefficients in eq 9 are obtained as first-order derivatives of the crystal field parameters *B*_*m*_^*l*^ with respect to the molecular Cartesian coordinates. They are then transformed into the crystal coordinate basis according to the following relation^5,8^
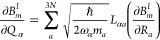
10where *Q*_α_ is the displacement vector corresponding to the normal mode α with angular frequency ω_α_, *N* is the number of atoms in the unit cell, *L*_*αa*_ is the Hessian matrix eigenvector, and *R*_*a*_ is the Cartesian degree of freedom. With the knowledge of the coupling coefficients, the relaxation rate for the transition between two spin eigenstates |*a*⟩ and |*b*⟩ is determined under the Born-Markov approximation by using the time evolution of the density matrix of the open quantum system. For the relaxation involving a single resonant phonon, the rate is^8,13^
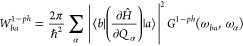
11where ℏω_*ab*_ = *E*_*a*_ – *E*_*b*_, and the function *G*^1-*ph*^ is expressed as
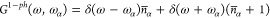
12where  is the Bose–Einstein distribution for the thermal population of phonons, *k*_B_ is the Boltzmann constant, and the first and second Dirac δ functions enforce the energy conservation during the absorption and emission of phonons, respectively. The temperature dependence of the spin-phonon relaxation rate arises through this thermal population of phonons and their energy distribution. For the two-phonon Raman relaxation, the transition rate is^8^
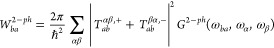
13where the function *G*^2–*ph*^ accounts for the process involving simultaneous absorption and emission of two phonons and is expressed as
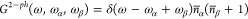
14where
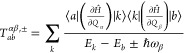
15The envelope of spin-excited states, |*k*⟩, that appears in eq 15 is commonly referred to as a virtual state to highlight the fact that such states do not get populated during Raman relaxation.^8^ Finally, the relaxation time (τ) is obtained by diagonalizing the matrix *W*_*ba*_^*n*–*ph*^ and taking the inverse of the smallest nonzero eigenvalue. τ obtained from *W*_*ba*_^1–*ph*^ provides the Orbach contribution to the total relaxation time (τ_Orbach_), whereas τ obtained from *W*_*ba*_^2–*ph*^ provides the Raman contribution to the total relaxation time (τ_Raman_). Finally, the total spin relaxation time τ due to the combined effect of both Orbach and Raman relaxation mechanisms can be obtained as .

## Computational Methods

For the three compounds investigated in this study, periodic density functional theory (pDFT), cell optimizations, and Γ-point phonon calculations were previously performed by Mondal et al.^8^ using the CP2K software.^56^ For these calculations, the experimental X-ray structures were used as starting points for geometry optimization, and the PBE functional^44^ with the inclusion of the D3 dispersion correction^57^ was employed. We used the same optimized molecular geometries and phonon modes for this work. Tests on the importance of the full integration of the Brillouin zone were performed previously for **2** and found that it only becomes slightly relevant at temperatures below 5 K. The electronic structure calculations were performed using OpenMolcas version 24.02.^58^ The second-order Douglas–Kroll–Hess (DKH) Hamiltonian was used to account for the scalar relativistic effects, along with all-electron ANO-RCC basis sets contracted to polarized triple-ζ quality (ANO-RCC-VTZP) for Co and Dy and double-ζ quality (ANO-RCC-VDZ) for the rest of the atoms. An active space (AS) of seven electrons in the five 3d orbitals (7e, 5o) is used for the Co(II)-compounds. For the Dy(III)-compound, two different active spaces were considered. The minimal AS consists of nine electrons in the seven 4f orbitals (9e, 7o). The second active space also includes a second shell of f orbitals (9e, 14o). For compounds **1** and **2**, a state-average CASSCF calculation was performed over all possible (10) quartet states. For compound **3**, a state average over all possible (21) sextet states was performed with the (9e, 7o) AS. CASPT2 and MC-PDFT calculations were carried out using these reference CASSCF wave functions. For **1** and **2**, SOC among all of the spin-free quartet states (among all of the spin-free sextet states in case of **3**) were incorporated through the atomic mean-field integral (AMFI) approximation implemented in the restricted active space state interaction (RASSI) module of OpenMolcas.^59^ Doublets for the Co(II) systems (doublets and quartets for the Dy(III) system) were excluded from the SOC calculations due to significant energy separation from the low-lying quartets (sextets for the Dy(III) system). The translated “on-top” PBE functional (“tPBE”)^23^ was used to compute the MC-PDFT energies.

From each *ab initio* calculation, the crystal field parameters *B*_*m*_^*l*^, which fully define the spin Hamiltonian of the system (eq 4) can be computed. These parameters are derived from the electronic Hamiltonian, *Ĥ*_*el*_, expressed in the spin-free basis, together with the spin and orbital angular momentum matrices *Ŝ*_*i*_ and *L̂*_*i*_, with *i* = *x*, *y*, *z*. The mapping shown in eq 5 is performed with the *get*_*CF.x* routine distributed with the MolForge software^13^ (freely available at github.com/LunghiGroup/MolForge). The MolForge software also allows the calculation of the Orbach and Raman relaxation times by implementing eqs 11 and 13. To set up the calculation, first the three terms of the Hamiltonian in eq 7 must be provided. The ground-state electronic part of the Hamiltonian, *Ĥ*_*s*_, is expressed as a list of crystal field parameters, *B*_*m*_^*l*^, generated by the routine *get*_*CF.x*. The phonon bath, *Ĥ*_*ph*_, is constructed from the Hessian matrix obtained with pDFT. The code internally extracts the Hessian matrix eigenvectors *L*_*αa*_ and their corresponding frequencies ω_α_, which are used in eq 10.

The spin-phonon coupling matrix elements in Cartesian coordinates are obtained through numerical differentiation of the crystal field parameters by a step of ±0.01 Å. These values are provided as input to MolForge, which internally transforms them in the basis of the crystal coordinates by using eq 10. Tests on the validity of the numerical approach employed are reported in section S010 of the Supporting Information (SI).

In addition to the total Hamiltonian, the user must specify the Euler angles linking the molecular reference framework (defined by the raw input atomic positions) to the framework, where the *z*-axis aligns with the magnetic easy axis of the system. This axis is defined as the principal eigenvector of the matrix *gg*^*T*^, where *g* is the 3 × 3 *g*-tensor of the first Kramers doublet and *g*^*T*^ its transpose. Defining the Euler angles is critical for applying a small magnetic field of ∼0.01 T along the easy axis of magnetization when computing Raman relaxation times, as this decouples the coherence and population density matrix elements.^13^

Finally, for a user-defined set of temperatures, the programs output the values of τ_Orbach_ and τ_Raman_. The role of temperature in these simulations is to modify the Bose–Einstein population term *n̅*_α_, which enters the expressions of the transition rates through *G*^1–*ph*^ and *G*^2–*ph*^ (eqs 12 and 14, respectively).

## Results and Discussion

### **A.** [Co(C_3_S_5_)_2_](Ph_4_P)_2_ (**1**)

The ground state of complex **1** is a *S* = 3/2 spin state, giving rise to 2 sets of Kramers doublets separated by 282 cm^–1^ at the CASSCF level. The CASPT2 and MC-PDFT methods slightly open up the gap to 303 and 309 cm^–1^, respectively. (See Table 1).

**Table 1:** Energies of the Lowest Kramers Doublets (in cm^–1^) for Complex **1**

states	CASSCF	CASPT2	MC-PDFT
KD_0_	0	0	0
KD_1_	282	303	309

Importantly, we observed that the numerical derivatives of the crystal field parameters, i.e., the spin-phonon coupling coefficients () are quite different between CASSCF and CASPT2 methods, whereas they are relatively consistent between the CASSCF and MC-PDFT methods as can be seen from the parity plots in Figure S1(a,b) respectively. On the other hand, the coupling coefficients differ significantly between the CASPT2 and MC-PDFT methods, although they provide very similar energy gaps between the KDs (Figure S2). This suggests that the coupling strengths do not have a one to one correspondence with the excitation energies from the ground to excited KDs.

**Figure 2:**
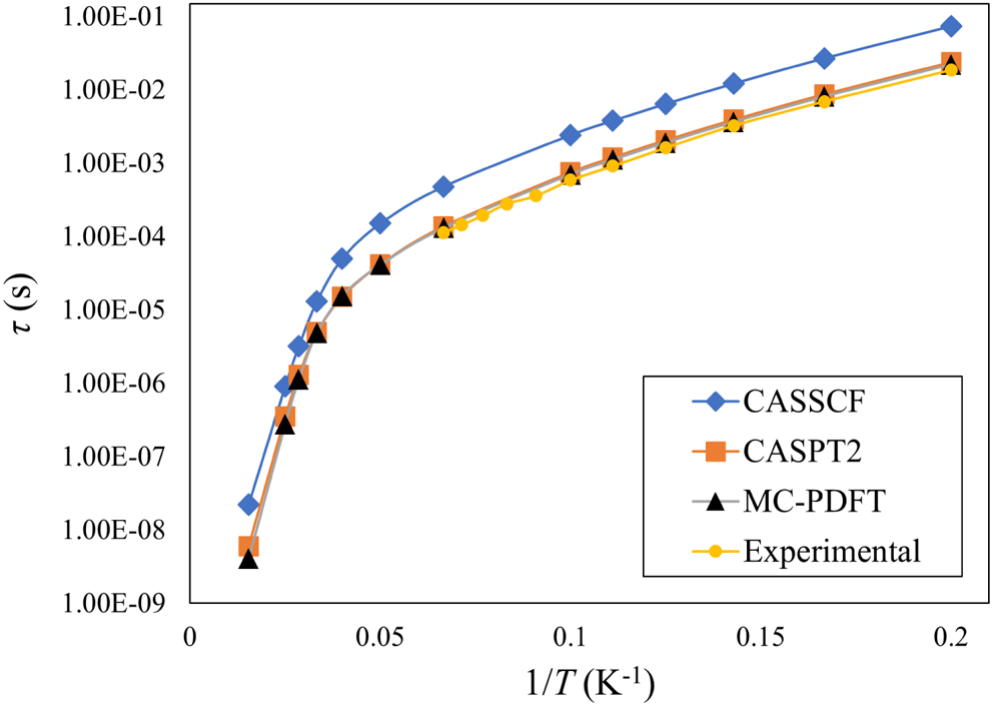
Total spin relaxation time (τ) as a function of 1/*T* for complex **1** obtained by different methods. Experimental data is taken from ref (36).

We then computed the Orbach and Raman relaxation rates using eqs 11 and 13, respectively, at different temperatures. The corresponding relaxation times (τ_Orbach_ and τ_Raman_) are obtained by taking the inverse of the rates. The total relaxation times τ at different temperatures computed with CASSCF, CASPT2, and MC-PDFT are reported in Table S1. The temperature dependence of τ obtained from these three methods along with the available experimental data for compound **1** is shown in Figure 2.

Although the overall trend in τ follows the experimental trend for all three methods, τ is overestimated by an order of magnitude at the CASSCF level compared to the experimental data, while CASPT2 and MC-PDFT provide very similar spin relaxation times and they both agree well with the experimental data. We could compare the relaxation time with the experimental data only in the low temperature region due to the unavailability of experimental data at relatively higher temperatures between 20 and 65 K (1/*T* between ∼0.07 and ∼0.02 K^–1^). An enlarged view of Figure 2 is provided in Figure S3, offering a clearer illustration of the agreement between CASPT2 and MC-PDFT predictions with the experimental results.

Despite differences in spin-phonon coupling coefficients between the two post-CASSCF methods, both yielded almost identical spin relaxation times for complex **1**. The reason is that, besides the coupling coefficient, other factors contribute to the rate expressions (eqs 11 and 13), namely, the spin eigenstates. The shape of the time versus 1/*T* curves thus reflects all of them.

### **B.** [CoL_2_][(HNEt_3_)_2_] (**2**)

Similar to complex **1**, the ground state of complex **2** has spin *S* = 3/2. The Kramers doublets are separated by 207 cm^–1^ at CASSCF, 254 cm^–1^ at CASPT2, and 191 cm^–1^ at MC-PDFT levels (Table 2). The spin-phonon parameters are reasonably consistent across all three methods—as illustrated in Figures S4 and S5. As for complex **1**, this suggests that the coupling strengths do not have a direct correspondence with the KD-excitation energies.

**Table 2:** Energies of the Lowest Kramers Doublets (in cm^–1^) for Complex **2**

states	CASSCF	CASPT2	MC-PDFT
KD_0_	0	0	0
KD_1_	207	254	191

**Figure 3:**
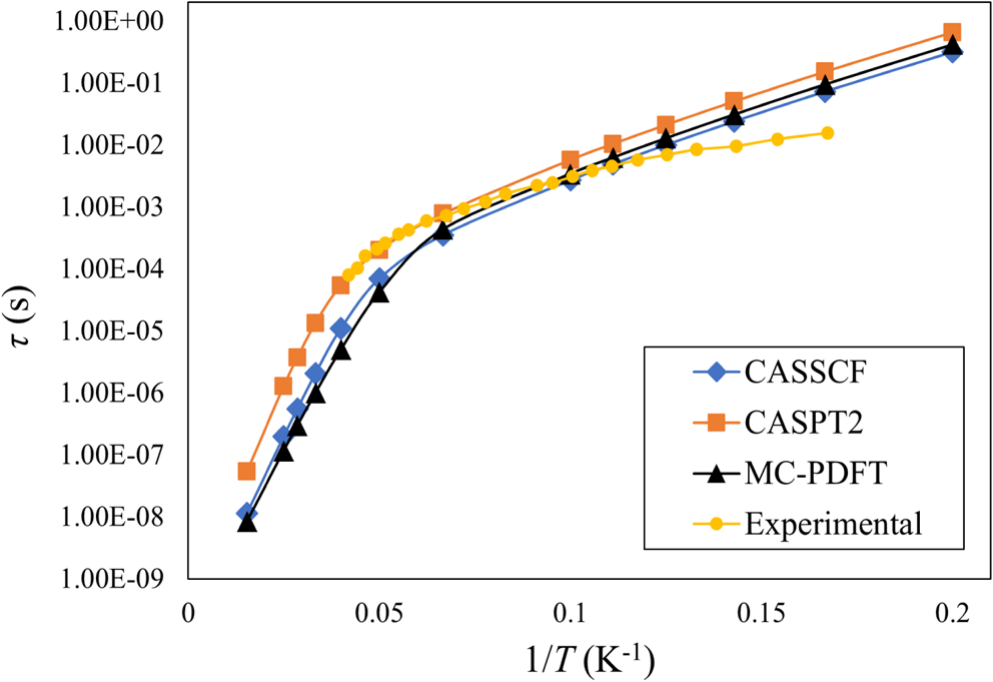
Total spin relaxation time (τ) as a function of 1/*T* for complex **2** obtained from different methods. Experimental data is taken from ref (37).

Figure 3 shows that τ obtained from CASPT2 overlaps with the experimental relaxation times in the temperature range between 15 and 20 K (1/*T* between 0.07 to 0.04 K^–1^), whereas CASSCF and MC-PDFT values are lower. An enlarged view of Figure 3 is provided in Figure S6, clearly illustrating the agreement between CASPT2 and experimental results. Between 5 and 10 K (1/*T* between 0.2 and 0.1 K^–1^), the deviation of all of the computed τ values from the experimental data arises due to the lack of consideration of the quantum tunneling of magnetization (QTM) mechanism of spin relaxation in our simulation, which appears to significantly contribute to the experimentally observed total spin relaxation. At relatively higher temperatures between 20 and 65 K (1/*T* from 0.05 to 0.01 K^–1^), the relaxation times predicted by CASSCF and MC-PDFT are in close agreement, while CASPT2 estimates slightly longer relaxation times, making them overlap with the experimental data within the range where the latter is available. This trend in the plot also suggests that an extrapolation of the CASPT2 predictions would continue to closely align with the experimental curve if it were available for that temperature range.

### **C.** [Dy(bbpen)Cl] (**3**)

**Figure 4:**
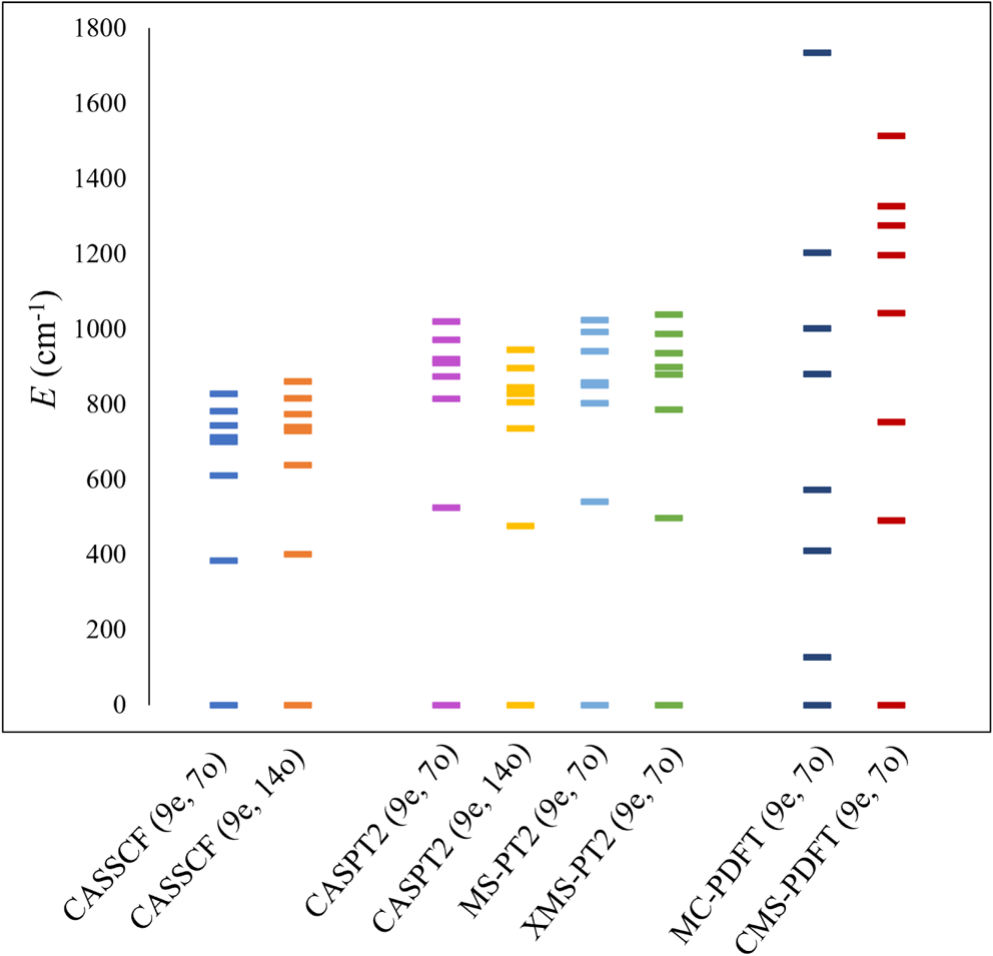
Energy spacings among the ground and excited Kramers doublets obtained from different methods for compound **3**. Active spaces employed for the calculations are mentioned in parentheses.

Complex **3** exhibits a ground-state multiplet ^6^*H*_15/2_ with total angular momentum *J* = 15/2, giving rise to 8 Kramers doublets. The energy spacings among these Kramers doublets obtained from different methods are shown in Figure 4. The absolute energies of all of the KDs computed by these methods are provided in Table S5.

**Figure 5:**
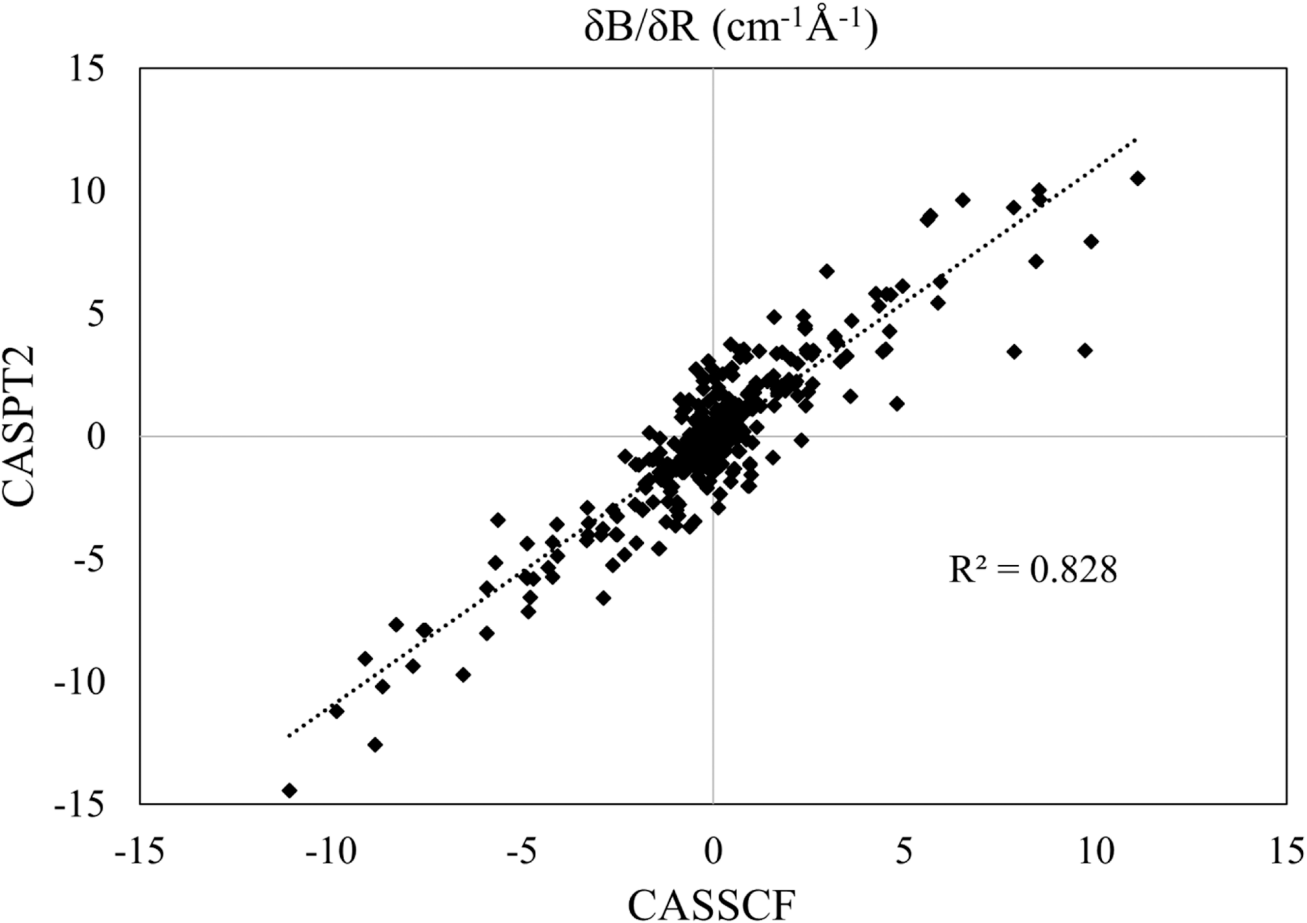
Parity plots comparing the numerical derivatives of the crystal field parameters computed at the CASSCF and CASPT2 levels for compound **3**.

We begin by focusing on the results obtained using the smaller active space (9e, 7o). Similar to complexes **1** and **2**, CASPT2 opens up the gap between the ground and first excited KD. The CASPT2 energy difference (ΔKD_0_^7^) between the ground KD (KD_0_) and the 7th excited KD (KD_7_) is 1020 cm^–1^ compared to the CASSCF value of 827 cm^–1^. On the other hand, with MC-PDFT, the first excited KD (KD_1_) is very close to KD_0_, unlike CASSCF and CASPT2, and ΔKD_0_^7^ is high in energy, 1734 cm^–1^. Due to the possible strong interaction among the closely spaced KDs, we also investigated the performance of the multistate versions of these methods, namely, multistate^60^ (MS), extended multistate^61,62^ (XMS) CASPT2, and compressed multistate PDFT (CMS-PDFT).^63^ MS-CASPT2 and XMS-CASPT2 give energy levels similar to those of CASPT2 (Figure 4). On the other hand, CMS-PDFT provides a much more consistent energy spacing among the lowest KDs as compared to the single-state MC-PDFT, although ΔKD_0_^7^ is still very large (1513 cm^–1^). The performance of MC-PDFT is highly dependent on the quality of the functional used. The tPBE on-top pair-density functional we used here may not be adequate enough to capture the intricate electron correlation in the partially filled and highly localized 4f orbitals of the Dy system. Importantly, the functionals used in MC-PDFT calculations are translations of KS-DFT functionals with *unoptimized* fixed parameters; e.g., in this work, we have employed the translated PBE (tPBE) exchange-correlation functional. Moreover, the tPBE functional is not “fully translated,” meaning only the gradient of total density was translated, and the translation of the gradient of the on-top pair density was not considered; this leaves some physics contained in the gradient quantity uncaptured. Both of these factors may lead to significant deviations from the results obtained using CASPT2, which provides a more accurate description, especially for systems with an overly complex electronic configuration such as that present in lanthanide complexes with partially filled f orbitals. This suggests that electron correlation beyond what MC-PDFT captures may be crucial for such strongly correlated 4f systems. This behavior has been previously detected for lanthanide and actinide compounds with dense energy levels,^64^ and new state interaction formulations of PDFT^65^ are under investigation. In the following, we will only discuss the CASPT2 results and compare them with previously reported CASSCF results.^38^ The spin-phonon coupling coefficients are rather consistent between these two methods (Figure 5).

**Figure 6:**
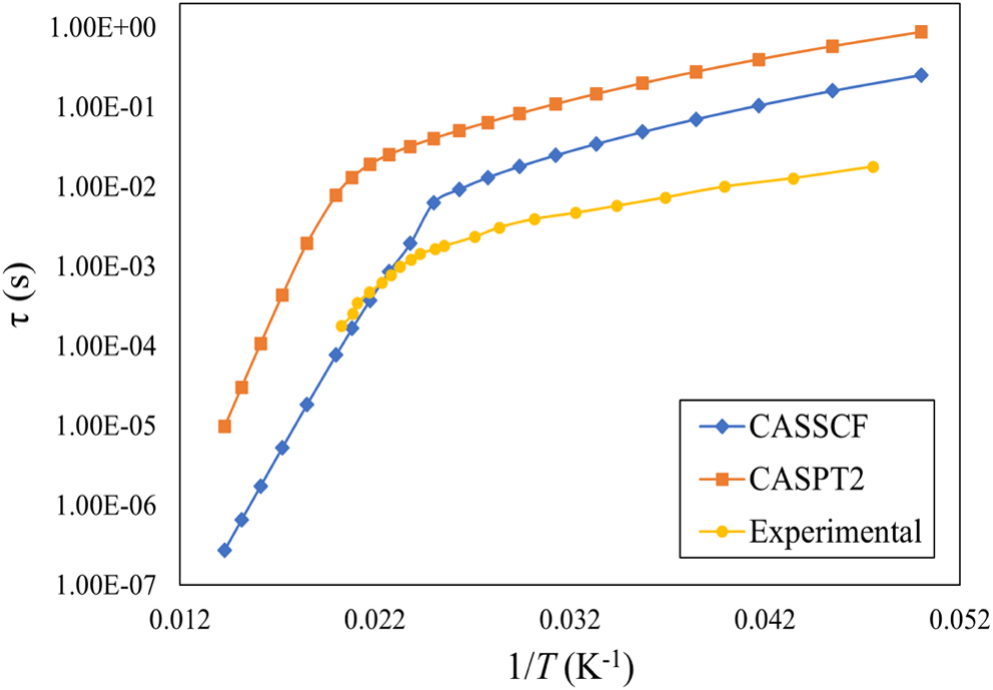
Total spin relaxation time (τ) as a function of 1/*T* for complex **3** obtained by different methods. Experimental data is taken from ref (38).

The overall trends of CASSCF and CASPT2 total relaxation time vs temperature reproduce the experimental trend (Figure 6). However, CASSCF and CASPT2 overestimate the experimental data by 1 and 2 orders of magnitude, respectively. One difference between the two methods is that CASPT2 predicts too high Kramers doublet energies compared to CASSCF. It has been previously reported for lanthanide systems that when a small active space is used, CASPT2 may predict too high crystal field splitting.^27^ Expanding the AS by means of a second shell of f orbitals may counteract this CASPT2 effect and provide more accurate crystal field splittings. For the equilibrium geometry, we tested the CASPT2 performance using the (9e, 14o) AS. We used as a guess the SA-CASSCF(9e, 7o) wave function and considered an averaging over the same number of states (21). We indeed observed a significant reduction in the crystal field splitting of the ground electronic state (Figure 4). However, the increase in computational cost associated with the larger AS restricts us to use it for the study of spin relaxation dynamics, especially since the CASSCF and CASPT2 need to be performed for all of the distorted structures to compute the numerical differentiation of the crystal field Hamiltonian.

## Discussion and Conclusions

CASSCF methods have been extensively used to simulate spin relaxation time as a function of the temperature of the phonons bath and have been shown to reproduce experimental trends.^8,13,66^ However, the systematic exploration of multiple molecules has shown that quantitative inaccuracies persist.^8^ In particular, it seems not currently possible to confidently rank the relaxation rate of different compounds unless they differ by at least 1 order of magnitude. The present study presents the first systematic exploration of multireference post-CASSCF methods, CASPT2 and MC-PDFT, for the simulations of spin relaxation in mononuclear single-molecule magnets, and the results show that the dynamical electron correlation beyond the active space is critical in explaining the discrepancies previously observed.

CASPT2 systematically improves the agreement between experiments and simulations and achieves quantitative accuracy for both Co compounds studied. Interestingly, this is observed both in cases where CASSCF underestimates or overestimates experimental results by 1 order of magnitude, suggesting that CASPT2 is able to capture nontrivial correlations between chemical structure, zero-field splitting, and spin-phonon coupling. MC-PDFT on the other hand matches the accuracy of CASPT2 for System **1** but retains the same level of error of CASSCF for System **2**. A detailed analysis of all of the computed quantities determining spin relaxation, i.e., the Hamiltonian eigenvalues, Hamiltonian eigenstates, and spin-phonon coupling coefficients, highlights the absence of a simple explanation for CASSCF’s deficiencies and that the improvement of the spin relaxation time at the CASPT2 level can only be tentatively ascribed to an overall simultaneous better accuracy for all of these quantities. Although larger benchmarks will be necessary to prove the generality of this important result, CASPT2 appears to achieve the goal of quantitative and systematic predictions for Co-based mononuclear compounds. Although no evidence of the importance of a full phonons Brillouin zone integration was found for molecule **2**, testing the robustness of this finding against this and other fine effects stands out as the next step forward.^11^

The situation is drastically different for the Dy compound (System **3**), where all computational methods deviate significantly from experiments and discrepancies span 1–2 orders of magnitude. Interestingly, our results suggest that the overall high-level and systematic accuracy observed in the prediction of spin-phonon relaxation times in Dy compounds with CASSCF might be partially due to a cancellation of errors. Indeed, here, we have shown that the inclusion of correlation beyond the active space through CASPT2 drastically decreases the accuracy of predictions. The use of a larger active space seems to alleviate this issue, but full convergence of results with respect to the active space size could not be achieved without incurring extensive computational costs.

The difficulty in converging the size of the active space for the Dy compound mostly comes from the large expense of computing spin-phonon coupling coefficients, which requires at least six times the number of atoms in CASPT2 calculations. Numerical approaches to reduce the expense of multireference calculations or the number of single-point calculations to compute spin-phonon coupling are an urgent necessity. On the former front, MC-PDFT stands out as a promising route, but our results show that further development is required to consistently achieve CASPT2 levels of accuracy. In terms of lowering the number of calculations to estimate spin-phonon coupling, two routes have been recently pursued. The use of analytical gradients has been proposed,^67^ but it currently lacks the contribution of spin–orbit coupling derivatives, which has been shown to lead to errors for both Co and Dy compounds,^54^ including System (**3**). To the best of our knowledge, the implementation of the analytical gradients of the spin–orbit coupling operators has never been pursued but represents an interesting avenue of investigation. On the other hand, analytical gradients are not available for all methods and all codes, calling for alternative numerical strategies. In this regard, machine learning methods offer a very promising alternative.^68^ Seminal steps have been pursued in this direction, and savings up to 80% in the computation of phonons and spin-phonon coupling have been demonstrated.^69^ We anticipate that the combination of these data-driven strategies with high-level multireference methods might hold the solution to the challenges evidenced in this work for Dy molecules.

In conclusion, we have provided the first systematic investigation of the role of electronic correlation beyond the active space in the prediction of spin relaxation of mononuclear coordination compounds Co(II) and Dy(III). Our results provide important evidence that quantitative predictions can be achieved for Co-based molecules by employing CASPT2, while they suggest that further development is necessary for the Dy compounds. We anticipate that the long-sought goal of quantitative predictions of spin-phonon relaxation times is finally within reach and could be achieved through further investment in the development and benchmarking of multireference electronic structure methods.

## Supporting Information Available

Parity plots comparing the numerical derivatives of crystal field parameters for complex **1** and **2**; crystal field parameters computed by different methods for all of the equilibrium geometries, total spin-phonon relaxation times computed by different methods at different temperatures for all three systems; absolute energies of the lowest Kramers doublets for complex **3**; enlarged portions of plots for temperature dependence of spin relaxation time for complexes **1** and **2**; 20 largest spin-phonon coupling parameters obtained from different methods for all three complexes, a discussion on the validity of numerical differentiation to compute the spin-phonon coupling for complex **1**; sample input for MolForge software (PDF)
