# Supplementary material for: Role of Electron Correlation beyond the Active Space in Achieving Quantitative Predictions of Spin-Phonon Relaxation
Source: J Chem Theory Comput. 2025 Mar 12;21(6):2829–38. doi: 10.1021/acs.jctc.4c01696 (PMC11948317; doi:10.1021/acs.jctc.4c01696)
Supplement: Supplementary file 1 — ct4c01696_si_001.pdf [file ct4c01696_si_001.pdf]

Supporting Information: The Role of Electron Correlation Beyond the Active Space in Achieving Quantitative Predictions of Spin-Phonon Relaxation

Soumi Haldar,† Lorenzo A. Mariano,‡ Alessandro Lunghi,*,‡ and Laura Gagliardi*,†

†*Department of Chemistry, Chicago Center for Theoretical Chemistry, University of Chicago, Chicago, IL 60637, USA.*

‡*School of Physics and AMBER Research Centre, Trinity College, Dublin 2, Ireland*

E-mail: lunghia@tcd.ie; lgagliardi@uchicago.edu

Contents

S01 Parity plots for spin-phonon coupling coefficients at different levels of theories for $[Co(C_3S_5)_2](Ph_4P)_2$ (complex **1**) **S-3**

S02 Total spin-relaxation time at different temperatures and crystal field parameters at different levels of theories for complex **1** **S-5**

S03 Enlarged τ vs 1/T plot for complex **1** **S-6**

S04 Parity plots for spin-phonon coupling coefficients at different levels of theories for $[CoL_2][(HNEt_3)_2]$ (complex **2**) **S-7**

S05 Comment on the observations in the parity plots for complex **1** and complex **2** . **S-8**

S06 Total spin-relaxation time at different temperatures and crystal field parameters at different levels of theories for complex **2** **S-9**

S07 Enlarged τ vs 1/T plot for complex **2** **S-10**

S08 Total energies of Kramers doublets, total spin-relaxation time at different temperatures, and crystal field parameters at different levels of theories for [Dy(bbpen)Cl] (complex **3**) **S-11**

S09 Twenty largest spin-phonon coupling parameters obtained from different methods for all three complexes **S-14**

S010 Numerical differentiation analysis **S-16**

S011 Sample Input for Spin-phonon Relaxation Simulation using MolForge Software . **S-20**

S01. Parity plots for spin-phonon coupling coefficients at different levels of theories for $[Co(C_3S_5)_2](Ph_4P)_2$ (complex 1)

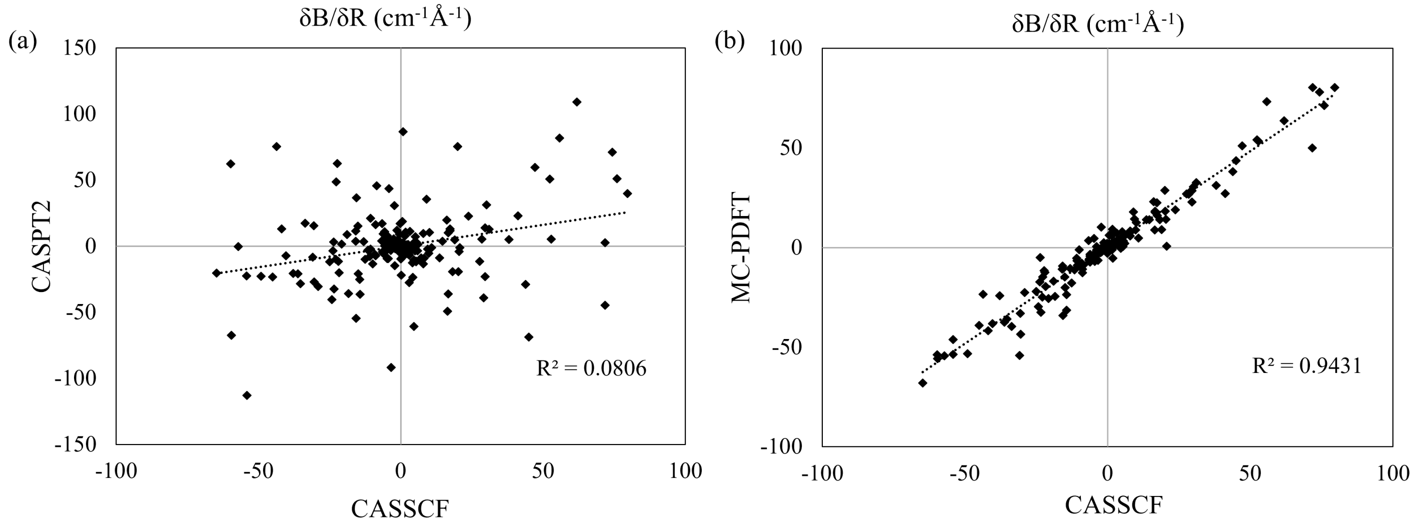

Figure S1: Parity plots comparing the numerical derivatives of the crystal field parameters computed at (a) CASSCF and CASPT2, and (b) CASSCF and MC-PDFT levels for compound **1**

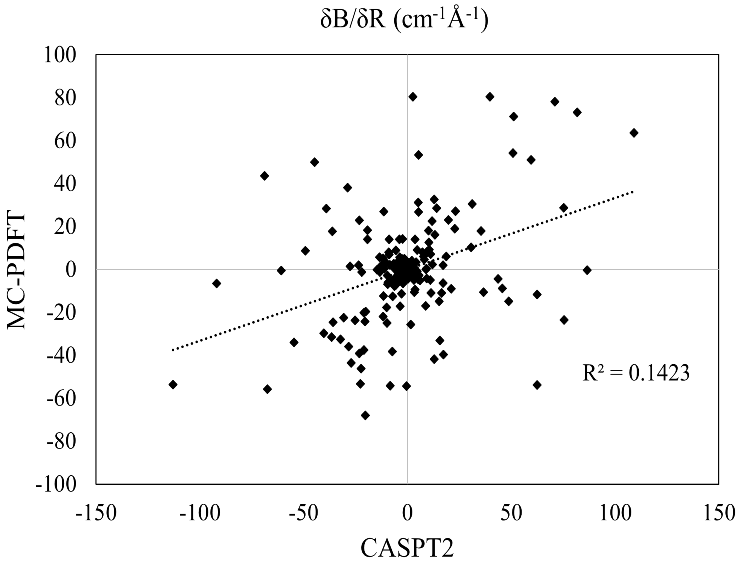

Figure S2: Parity plots comparing the numerical derivatives of the crystal field parameters computed at CASPT2 and MC-PDFT levels for compound **1**

S02. Total spin-relaxation time at different temperatures and crystal field parameters at different levels of theories for complex 1

Table S1: **Total (Raman and Orbach) spin-phonon relaxation time (in s) for complex 1 at different temperatures (in K)**

T(K)	CASSCF	CASPT2	MC-PDFT
65	2.23E-08	5.99E-09	4.04E-09
40	9.14E-07	3.51E-07	2.76E-07
35	3.20E-06	1.30E-06	1.12E-06
30	1.32E-05	4.96E-06	4.89E-06
25	4.99E-05	1.52E-05	1.56E-05
20	1.51E-04	4.16E-05	4.12E-05
15	4.82E-04	1.38E-04	1.32E-04
10	2.42E-03	7.53E-04	7.06E-04
9	3.79E-03	1.20E-03	1.12E-03
8	6.43E-03	2.06E-03	1.92E-03
7	1.22E-02	3.92E-03	3.66E-03
6	2.69E-02	8.70E-03	8.11E-03
5	7.36E-02	2.39E-02	2.23E-02

Table S2: **Crystal field parameters with rank l=2 for complex 1 obtained from different electronic structure methods**

**l**	**m**	**CASSCF**	**CASPT2**	**MC-PDFT**
2	-2	-37.4815997427	-39.6480873840	-38.8400812171
2	-1	-81.0118814590	-86.8943983279	-89.2846746409
2	0	-61.8173862193	-68.5556938298	-70.7614138596
2	1	-35.7200164846	-36.7863096775	-37.0155064971
2	2	14.2842230902	10.7275325245	8.36173272654

S03. Enlarged τ vs 1/T plot for complex 1

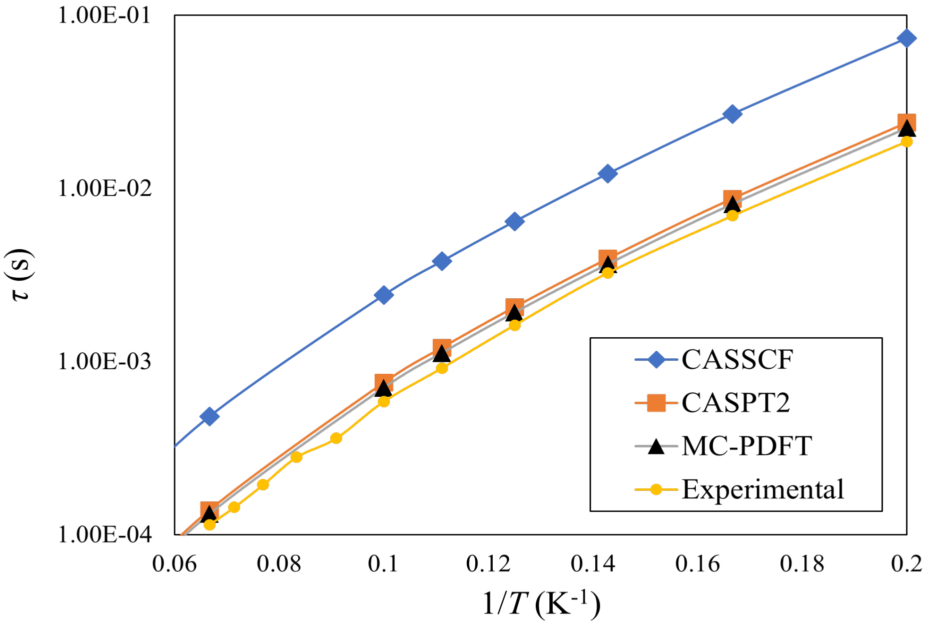

Figure S3: Enlarged portion of the total spin relaxation time as a function of 1/T for complex **1** obtained from different methods. Only the portion where experimental data is available is shown.

S04. Parity plots for spin-phonon coupling coefficients at different levels of theories for $[CoL_2][(HNEt_3)_2]$ (complex 2)

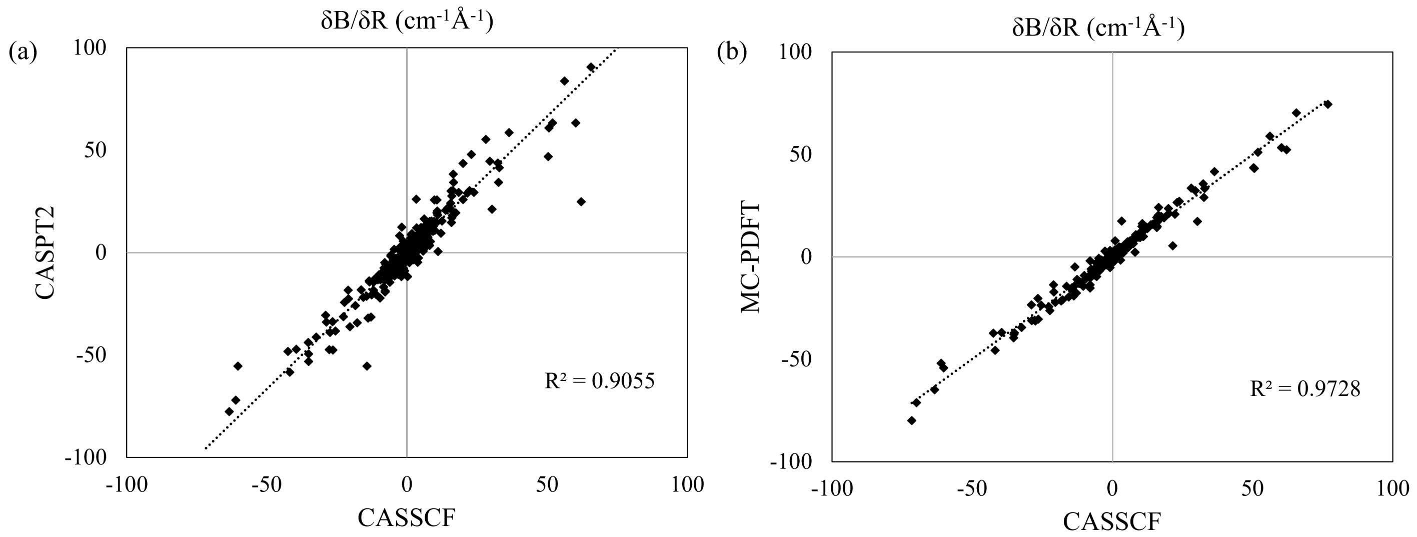

Figure S4: Parity plots comparing the numerical derivatives of the crystal field parameters computed at (a) CASSCF and CASPT2, and (b) CASSCF and MC-PDFT levels for compound **2**

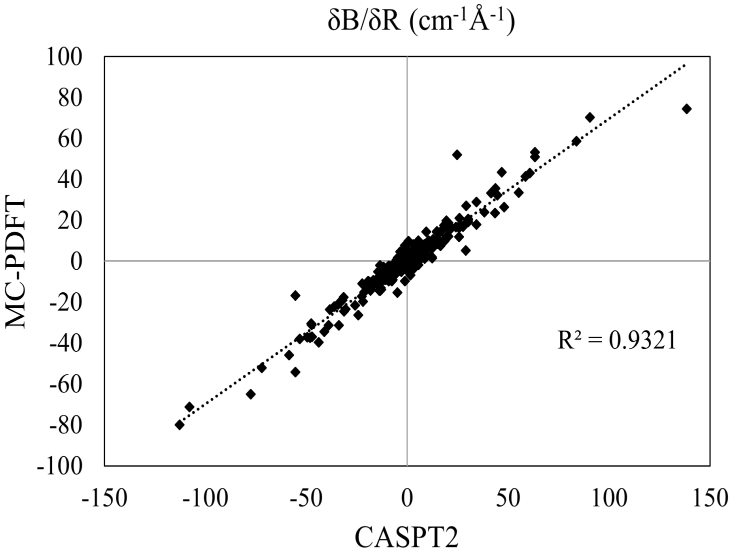

Figure S5: Parity plots comparing the numerical derivatives of the crystal field parameters computed at CASPT2 and MC-PDFT levels for compound **2**

S05. Comment on the observations in the parity plots for complex 1 and complex 2

The discrepancy observed in the parity plots of the spin-phonon coupling coefficients at different levels of theories may arise due to several factors. The direct interpretation of the significant deviations of the CASPT2 couplings from CASSCF/MC-PDFT ones for complex 1, but not so much for complex 2 is that the dynamic correlation introduced by CASPT2 in complex 1 leads to higher sensitivity of the crystal field parameters to nuclear displacements - potentially causing the observed deviation in the coupling coefficients. The structural sensitivity of CFPs in complex 2 is not as much as it is in complex 1. This is most likely due to the different symmetry and ligand field or coordination environment in the two complexes, which directly influences the CFPs and their response to nuclear displacements. Overall, a complex interplay of all these factors contributes to the observed deviations for complex 1 but not for complex 2.

S06. Total spin-relaxation time at different temperatures and crystal field parameters at different levels of theories for complex 2

Table S3: **Total (Raman and Orbach) spin-phonon relaxation time (in s) for complex 2 at different temperatures (in K)**

T(K)	CASSCF	CASPT2	MC-PDFT
65	1.14E-08	5.39E-08	8.40E-09
40	1.98E-07	1.28E-06	1.16E-07
35	5.54E-07	3.74E-06	2.97E-07
30	2.07E-06	1.34E-05	1.00E-06
25	1.10E-05	5.44E-05	5.03E-06
20	6.91E-05	2.02E-04	4.17E-05
15	3.52E-04	7.76E-04	4.36E-04
10	2.69E-03	5.75E-03	3.42E-03
9	4.89E-03	1.04E-02	6.25E-03
8	9.95E-03	2.11E-02	1.28E-02
7	2.39E-02	5.04E-02	3.10E-02
6	7.24E-02	1.52E-01	9.48E-02
5	3.16E-01	6.58E-01	4.17E-01

Table S4: **Crystal field parameters with rank l=2 for complex 2 obtained from different electronic structure methods**

**l**	**m**	**CASSCF**	**CASPT2**	**MC-PDFT**
2	-2	52.3513027859	65.2406010149	48.3073109264
2	-1	5.5939798322	5.4241423133	5.4531598212
2	0	39.6536082387	43.3833864989	37.7309149685
2	1	-10.6284364134	-13.4242802701	-9.6607835435
2	2	-51.5938862487	-66.5429870282	-46.9908292270

S07. Enlarged τ vs 1/T plot for complex 2

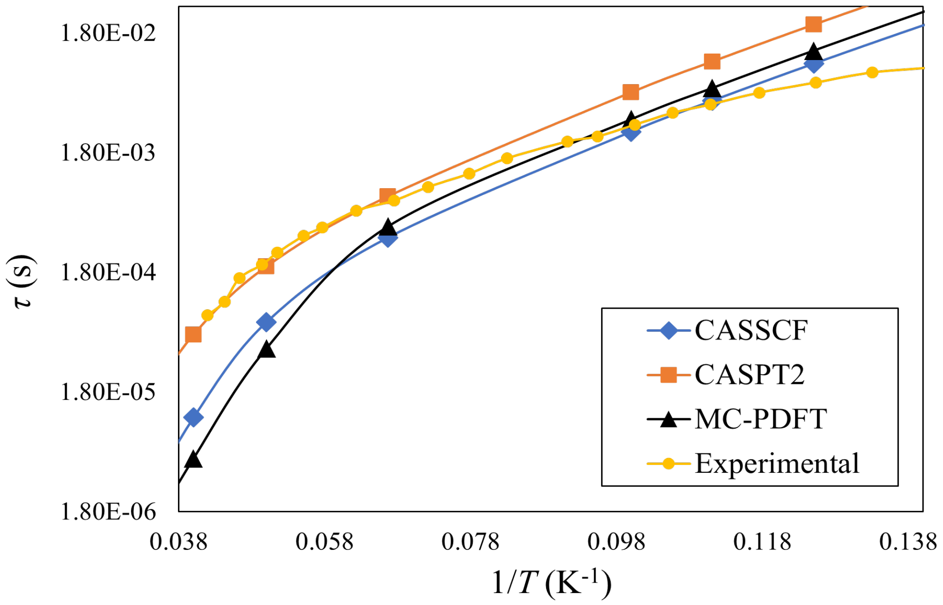

Figure S6: Enlarged portion of the total spin relaxation time as a function of 1/T for complex **2** obtained from different methods.

S08. Total energies of Kramers doublets, total spin-relaxation time at different temperatures, and crystal field parameters at different levels of theories for [Dy(bbpen)Cl] (complex 3)

Table S5: **Energies of the lowest Kramers doublets (in cm^{-1}) for complex 3 obtained from different electronic structure methods**

**States**	CASSCF	CASSCF	CASPT2	CASPT2	MS-PT2	XMS-PT2	MC-PDFT	CMS-PDFT
AS	(9e, 7o)	(9e, 14o)	(9e, 7o)	(9e, 14o)	(9e, 7o)	(9e, 7o)	(9e, 7o)	(9e, 7o)
KD_0	0	0	0	0	0	0	0	0
KD_1	383	401	524	476	540	497	127	490
KD_2	610	638	814	735	802	785	410	752
KD_3	700	728	873	805	850	879	572	1042
KD_4	711	740	909	827	858	898	880	1195
KD_5	743	774	919	844	941	935	1001	1275
KD_6	781	816	971	896	992	986	1202	1326
KD_7	827	861	1020	945	1023	1038	1734	1513

Table S6: **Total (Raman and Orbach) spin-phonon relaxation time (in s) for complex 3 at different temperatures (in K)**

T(K)	CASSCF	CASPT2
70	2.76E-07	9.83E-06
66	6.59E-07	3.04E-05
62	1.76E-06	1.08E-04
58	5.31E-06	4.37E-04
54	1.86E-05	1.97E-03
50	7.72E-05	7.83E-03
48	1.68E-04	1.31E-02
46	3.78E-04	1.92E-02
44	8.65E-04	2.54E-02
42	1.94E-03	3.21E-02
40	6.29E-03	4.04E-02
38	9.30E-03	5.07E-02
36	1.31E-02	6.44E-02
34	1.80E-02	8.31E-02
32	2.48E-02	1.09E-01
30	3.45E-02	1.46E-01
28	4.88E-02	1.99E-01
26	7.06E-02	2.77E-01
24	1.05E-01	3.96E-01
22	1.60E-01	5.79E-01
20	2.53E-01	8.74E-01

Table S7: **Crystal field parameters with rank l=2,4, and 6 for complex 3 obtained from different electronic structure methods**

l	m	CASSCF	CASPT2	MC-PDFT
2	-2	-0.0602808563	-0.0584151270	1.0100293501
2	-1	-0.0771908521	-0.0868214284	-0.2539106822
2	0	-8.3953984952	-9.8822645084	-12.0538479929
2	1	-5.3895729295	-6.3163265970	-1.3350032254
2	2	-1.0807291113	-1.7549146336	2.0351924303
4	-4	0.0000602463	0.0001866959	-0.0755734625
4	-3	-0.0001145330	-0.0006822947	0.0061446938
4	-2	-0.0012375510	-0.0018219792	0.0169027173
4	-1	-0.0002056831	-0.0006683095	0.0140128842
4	0	-0.0556293157	-0.0926722574	-0.0763703710
4	1	-0.1259640537	-0.1689093447	0.1538173692
4	2	-0.1011952665	-0.1437226204	-0.0320265922
4	3	-0.0051266162	-0.0286957059	-0.0026224074
4	4	-0.0115277755	-0.0149133951	0.1498781579
6	-6	0.0000061584	0.0000072238	0.0021639721
6	-5	0.0000080374	0.0000087949	-0.0026910210
6	-4	0.0000015144	-0.0000029487	-0.0023455178
6	-3	-0.0000047578	-0.0000299542	0.0001066387
6	-2	-0.0000047095	-0.0000166701	-0.0021024178
6	-1	-0.0000026796	0.0000005354	-0.0001295590
6	0	-0.0019300881	-0.0030588908	0.0149679615
6	1	0.0006199017	0.0005692751	0.0046844970
6	2	-0.0002360301	-0.0005816410	0.0071353011
6	3	-0.0007688559	-0.0011013375	0.0007403851
6	4	0.0003874627	0.0005579542	0.0037013129
6	5	-0.0006828663	-0.0003500784	0.0046691693
6	6	0.0011446966	0.0009981620	-0.0038816405

S09. Twenty largest spin-phonon coupling parameters obtained from different methods for all three complexes

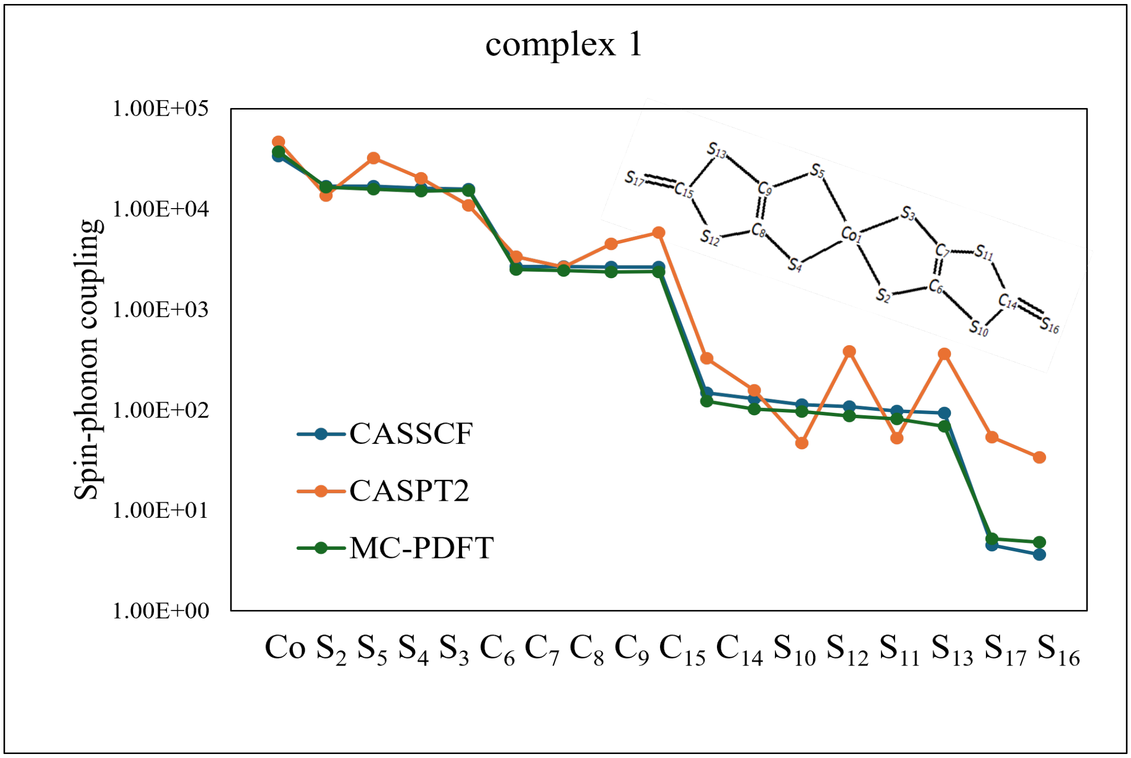

Figure S7: 17 largest spin-phonon coupling parameters for complex **1** obtained from different methods. The molecule with the atom indices is shown in the inset.

Fig. S7, S8 and S9 present the top 20 (top 17 for complex **1**) largest spin-phonon coupling values i.e. the derivatives of the spin Hamiltonian parameters with respect to the Cartesian degrees of freedom obtained using the CASSCF, CASPT2, and MC-PDFT methods. At the CASSCF level, we compute spin-phonon coupling for each atom by summing the squared coupling values for displacements along the x, y, and z directions. The atoms are then ranked in descending order based on these values. We maintain the same ranking when analyzing the CASPT2 and MC-PDFT results, allowing for direct comparison across different methods. We found that CASPT2 generally yields larger coupling values compared to CASSCF, and MC-PDFT. Although, the overall trends in coupling strengths predicted are quite similar across all the methods, with CASSCF and MC-PDFT data following each

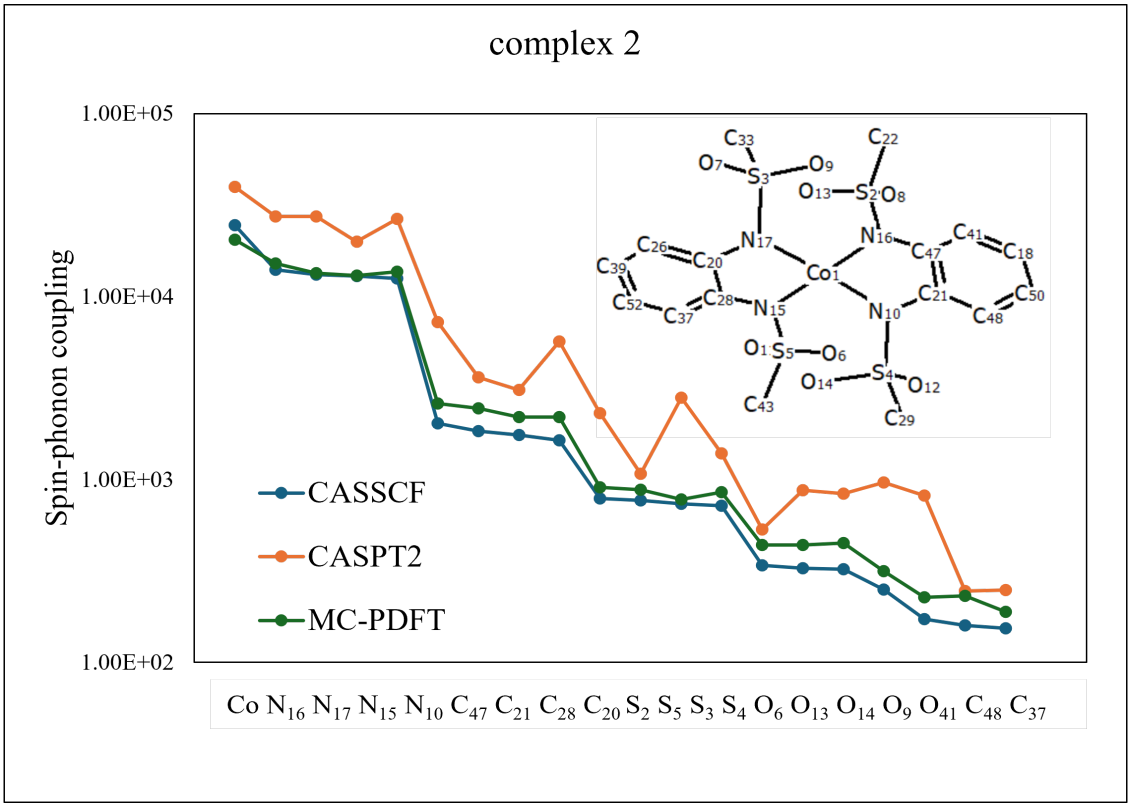

Figure S8: 20 largest spin-phonon coupling parameters for complex **2** obtained from different methods. The molecule with the atom indices is shown in the inset.

other very closely for complex **1** and complex **2**. This suggests that the dominant phonon modes that affect the spin-phonon relaxation most significantly, are consistent in all the electronic structure methods employed in this work, despite slight quantitative differences in the coupling strengths. Moreover, a systematic enhancement of the interactions between the spin-states of the system and the phonons are observed when dynamic correlation is included at the CASPT2 level.

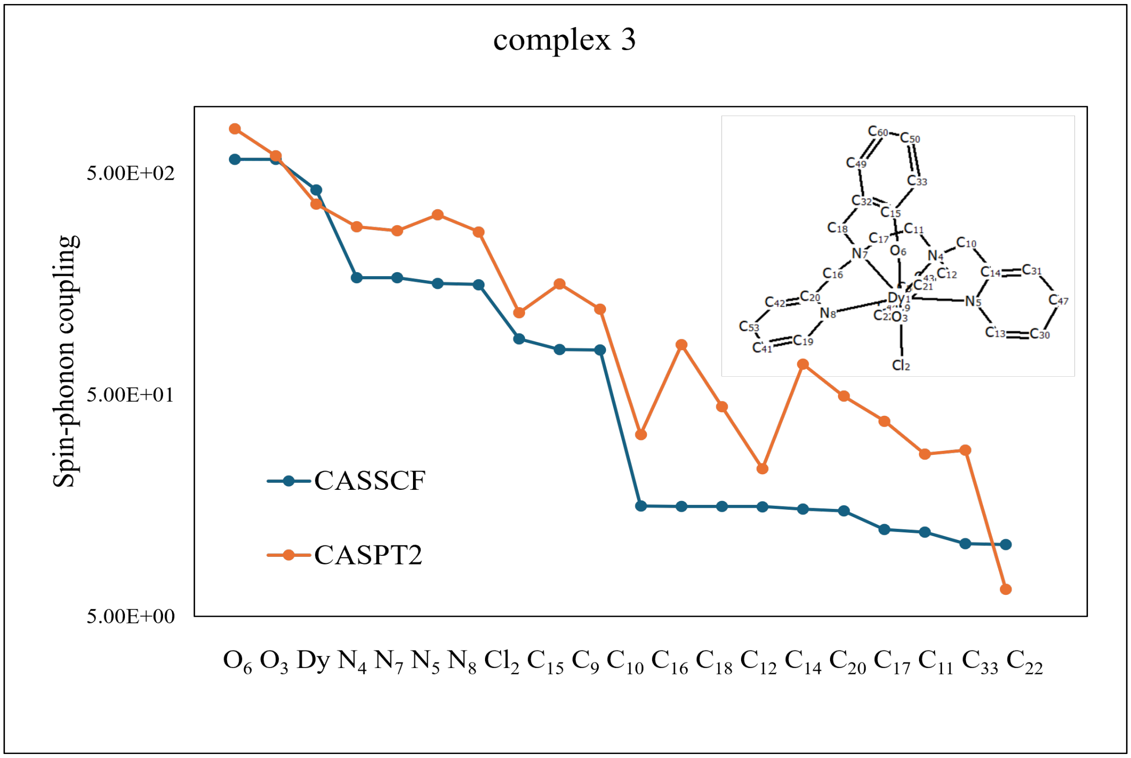

Figure S9: 20 largest spin-phonon coupling parameters for complex **3** obtained from different methods. The molecule with the atom indices is shown in the inset.

S010. Numerical differentiation analysis

Figures S10, S11, and S12 show the crystal field parameters for different displaced geometries computed by CASSCF, CASPT2, and MC-PDFT methods, respectively, for a selected degree of freedom of compound **1**. The results presented in this paper were obtained using a 2-points fitting around the equilibrium geometry with a differentiation step of 0.01 Å. This approach is justified by the smooth dependence of the crystal field parameters on geometrical displacements. However, CASPT2 calculations display slightly larger numerical noise compared with the other methods. To account for this, we recalculated the relaxation times for compound 1 using the CASPT2 method with both a 8-points fit and a hybrid approach. In the latter, the 2-point differentiation was refined by incorporating a larger fitting range when a poor linear trend was detected. These results are presented in Figure S13. The differences in spin-relaxation time obtained using different fitting procedures remain well within the

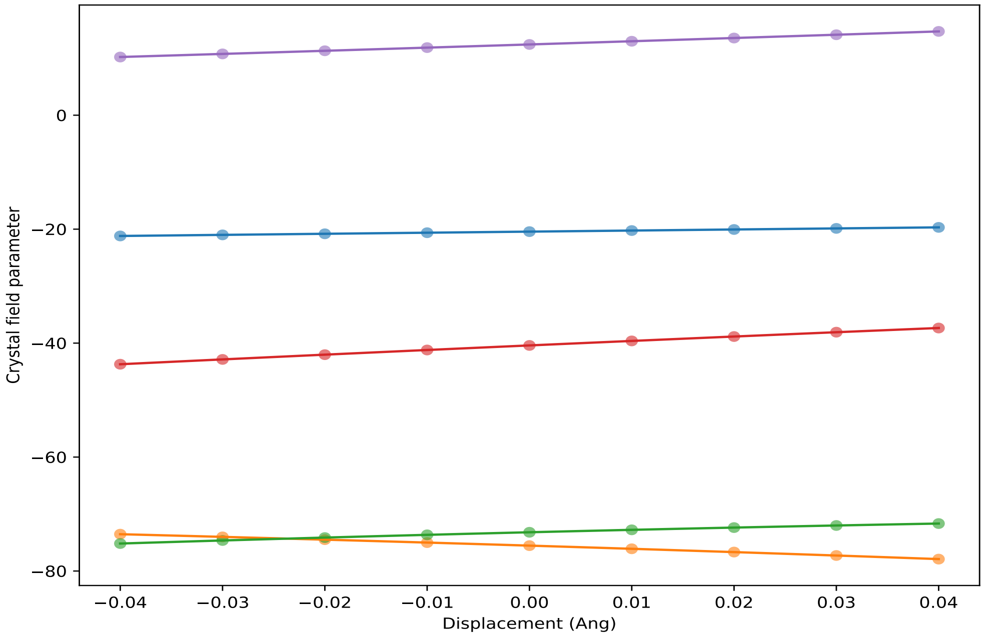

Figure S10: Computed crystal field parameters for compound **1** for different displacements around the equilibrium geometry of the Co atom along the x-axis using the CASSCF method. Color code: B^2_{-2} blue, B^2_{-1} orange, B^2_0 green, B^2_1 red, and B^2_2 purple.

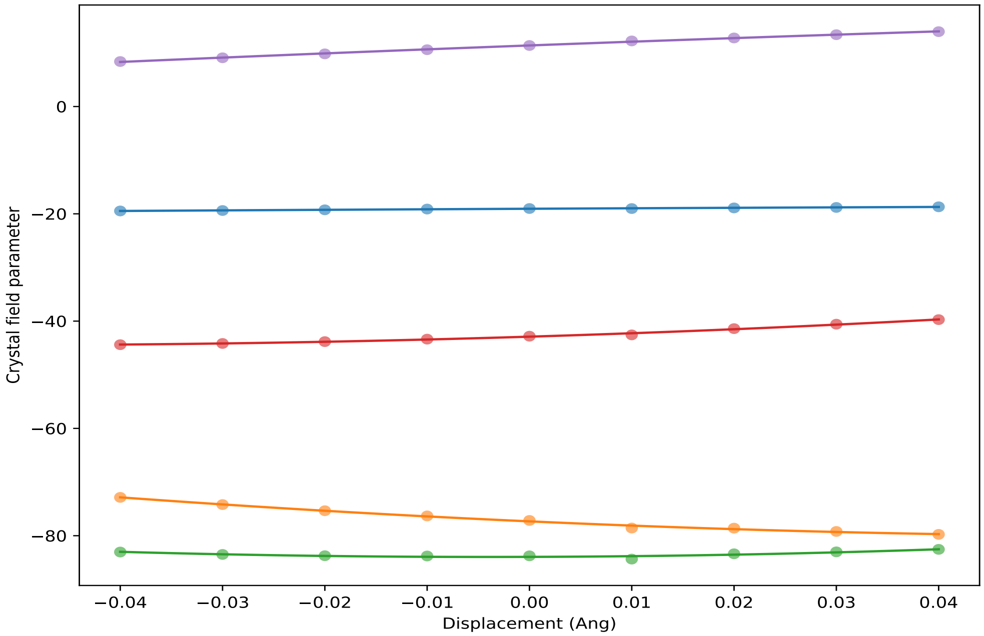

Figure S11: Computed crystal field parameters for compound **1** for different displacements around the equilibrium geometry of the Co atom along the x-axis using the CASPT2 method. Color code: B^2_{-2} blue, B^2_{-1} orange, B^2_0 green, B^2_1 red, and B^2_2 purple.

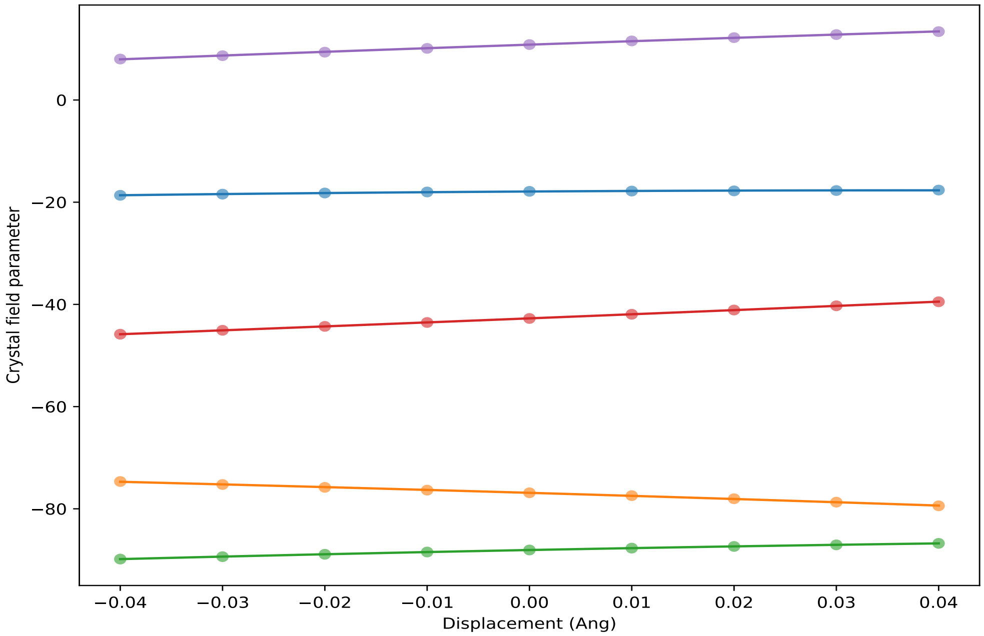

Figure S12: Computed crystal field parameters for compound **1** for different displacements around the equilibrium geometry of the Co atom along the x-axis using the MC-PDFT method. Color code: B^2_{-2} blue, B^2_{-1} orange, B^2_0 green, B^2_1 red, and B^2_2 purple.

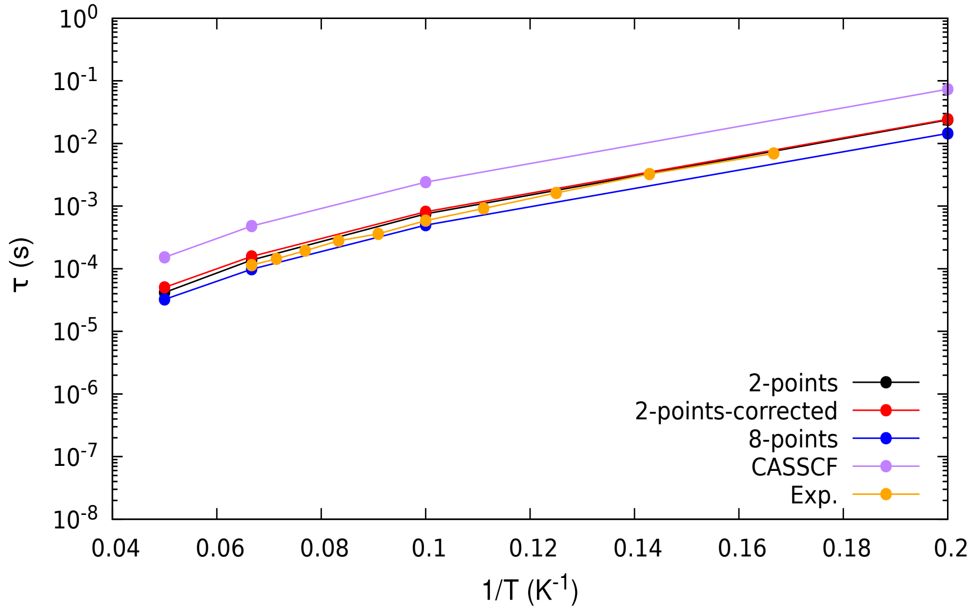

Figure S13: Computed relaxation time using the CASPT2 method for compound **1**: 2-point fit as in the main paper (black dots), 2-point fit with corrected outliers (red dots), and 8-point fit (blue dots). For reference, CASSCF data (purple dots) and experimental results (orange dots) are also included.

typical errors associated with this type of calculation and most importantly is smaller than the deviation between CASSCF and CASPT2. This confirms that the small numerical noise associated to the numerical calculation of spin-phonon coupling does not lead to significant variations in the computed spin-relaxation times.

S011. Sample Input for Spin-phonon Relaxation Simulation using MolForge Software

A sample input for the MolForge Spiral.x module used for the complex **1** is provided below. An elaborate description of the keywords can be found in MolForge manual (available at github.com/LunghiGroup/MolForge).

```
&SPIN_H
&DEF_G 1
2.0 0.0 0.0
0.0 2.0 0.000
0.0 0.0000 2.0
&END
&DEF_O 1 2
-2 -20.432430297018943
-1 -75.542676238271810
0 -73.199339681285309
1 -40.405904383210917
2 12.417022878429290
&END
EULER 1.0837654909188834
0.60154069129323695
2.6867033198157317
&END
&SYSTEM
B 0.0000 0.0000 0.3000
&DEF_SPINS
S 1 1.5 -0.466867723
```

&END

&CELL

A 100.00000 0.00000 0.00000

B 0.00000 100.00000 0.00000

C 0.00000 0.00000 100.00000

NREP 1 1 1

&COORD

S 1 7.84704255057099

14.39220222767899

5.00741750010644

&END

&END

&END

&SPH_H

&PHONDY

TEMP 25

K_MESH 1 1 1

SMEAR 25

SMEAR_TYPE 1

FC2 FC2

MAX_ENER 3500

MIN_ENER 8

&END

&O_BATH 1 2

FILENAME B_final.txt

NORDER 1

&END

```
SECULAR
PT2
&END
&HILBERT_SPACE
fulldiag
max_ex -1
max_corr -1
max_dist 100000.0
dump_freq 1
dump_s T
dump_mi 1 1
dump_rmat
&END
&DENSITY_MATRIX
TYPE FULLY_POLARIZED
&END DENSITY_MATRIX
BUILD_PROPAGATOR 1.00 1
PROPAGATE 50000
```
